# The diagnostic performance of chest radiographs for lung malignancy in symptomatic primary-care populations: A systematic review and meta-analysis

**DOI:** 10.1259/bjro.20210005

**Published:** 2021-07-29

**Authors:** Louis Dwyer-Hemmings, Cassandra Fairhead

**Affiliations:** 1 Institute of Medical and Biomedical Education, St George’s University, London, United Kingdom; 2 Guys’ and St Thomas’ NHS Foundation Trust, London, United Kingdom

## Abstract

**Objectives:**

To synthesise existing evidence for the diagnostic accuracy of chest radiographs to detect lung malignancy in symptomatic patients presenting to primary care.

**Methods:**

A systematic review was performed and reported in accordance with the PRISMA framework, using a protocol prospectively registered with the PROSPERO database (CRD42020212450). Nine databases were searched for relevant studies. Data were extracted and chest radiograph sensitivity and specificity calculated where possible. Risk of bias was assessed using a validated tool. Random effects meta-analysis was performed.

**Results:**

Ten studies were included. Sensitivity meta-analysis was performed in five studies which were not the high risk of bias, with summary sensitivity of 81% (95% CI: 74–87%). Specificity could be calculated in five studies, with summary specificity of 68% (95% CI: 49–87%).

**Conclusions:**

The sensitivity of chest radiographs for detecting lung malignancy in primary care is relatively low. Physicians and policymakers must consider strategies to attenuate the possibility of false reassurance with a negative chest radiograph for this significant pathology. Options include widening access to cross-sectional imaging in primary care; however, any intervention would need to take into account the medical and financial costs of possible over-investigation. Prospective trials with long-term follow-up are required to further evaluate the risks and benefits of this strategy.

**Advances in knowledge:**

The chest radiograph has a sensitivity of 81% and specificity of 68% for lung malignancy in a symptomatic primary-care population. A negative chest radiograph does not exclude lung cancer, and physicians should maintain a low threshold to consider specialist referral or cross-sectional imaging.

## Introduction

Malignancy is the second largest cause of mortality worldwide, and malignancy of the lung is the leading cause of cancer mortality.^1^ In the UK, lung malignancy is the third most common malignancy, with an estimated 130 new cases diagnosed each day, and causing approximately 35,300 deaths per year. Most lung malignancies are diagnosed as emergency presentations, with around three-quarters late stage at diagnosis. Where lung malignancy is identified at an early stage, it is most commonly the result of a ‘two-week wait’ referral (TWR) from primary care and is associated with improved outcomes.^2^ Thus, early detection in primary care is an essential strategy to reduce lung cancer mortality through early identification and an increased possibility of curative intervention.^3^

The chest radiograph is a key investigation in this approach, as National Institute of Clinical Evidence (NICE) referral guidelines state that any suspicious chest radiograph should provoke a TWR.^4^ Systematic review methodology has previously been utilised to investigate the sensitivity of chest radiographs in symptomatic patients,^5^ but to date there has been no systematic review assessing diagnostic accuracy of chest radiograph in a primary-care population. A directed investigation is warranted as difference in disease prevalence between primary and secondary care affects test performance.^6^ Furthermore, this study uses meta-analytic methodology to produce a quantitative summary estimate of chest radiograph sensitivity.

This study aims to provide an accurate estimation of the performance of the chest radiograph for detecting lung malignancy in a symptomatic primary-care population. This will assist primary-care physicians, when combined with their pre-test suspicion of malignancy, in deciding which patients require further imaging or referral. Furthermore, it will provide an evidence base for population health researchers and policymakers to formulate and review strategies to improve malignancy diagnosis and management on a national scale.

## Methods and materials

A systematic review methodology was performed and reported in accordance with the preferred reporting items for systematic reviews and meta-analyses (PRISMA) framework (see supplementary material online for full PRISMA checklist). The study was performed following a predefined protocol prospectively registered with the PROSPERO database (CRD42020212450). A search strategy was formulated and executed in the following nine databases:

- Allied and Complementary Medicine Database (AMED)
- British Nursing Index (BNI)
- Cumulative Index to Nursing and Allied Health Literature (CINAHL)
- EMBASE
- EMCare
- Health Management Information Consortium (HMIC)
- Medical Literature Analysis and Retrieval System Online (MEDLINE)
- PsycINFO
- PubMed

The search strategy used free-text words for the concepts of ‘chest radiograph, ‘lung malignancy’, ‘primary care’ and ‘diagnosis’. The full search strategy is reproduced in Supplementary Material 1. No linguistic or temporal limits were imposed, to access the broadest possible range of evidence for screening. A further manual search was performed using institutional websites to identify any relevant grey literature.^7–17^ Titles and abstracts of all studies were screened for relevance. A low threshold was used to include studies for full-text assessment. Article review was performed for potentially relevant studies with reference to inclusion and exclusion criteria to determine final selection. Screening and article review were performed independently by two authors, with any difference resolved through discussion and consensus. Finally, footnotes of included articles were reviewed to identify any further potentially relevant studies.

Studies were eligible if they reported a number of chest radiographs performed in primary care for symptomatic patients, a number of chest radiographs reported as ‘positive’ (any abnormality suspicious for malignancy identified) and a number of primary lung malignancies diagnosed pathologically or radiologically, as these are the minimum factors to calculate sensitivity. Studies that included data from primary-care settings alongside other settings were included if the primary-care population could be identified and analysed in isolation based on the information reported. Studies investigating patients aged over the 16 were included to maximise external validity and ensure that the majority of cases of malignancy were identified, although malignancy is more common in older demographics.

Studies were excluded if they used radiographs for screening, surveillance or staging; if populations were not representative (*e.g.,* if the patient population was chosen focussing on a specific risk factor, or pathological subtype of lung malignancy); or if they only reported data for non-primary lung cancers. Case reports were also excluded.

Data were extracted using a standardised proforma by a single author to improve standardisation. Variables extracted included title, year of publication and journal, setting, study design, population, and data regarding number of chest radiographs performed, positive chest radiographs, reference test used, and primary lung malignancies identified. Risk of bias for each included study was assessed using the QUADAS-2 tool. As the index test outcome was binary – either cross-sectional imaging or cytopathology which identified a malignancy or did not – the signalling question relating to threshold values was removed to tailor the tool to this review.^18^ This was performed independently by two reviewers, and any disagreement resolved through consensus.

Sensitivity of chest radiograph was calculated for each study using numbers of chest radiographs performed, number of radiographs reported as ‘positive’ for suspected lung malignancy and total number of primary lung malignancies in the population. Specificity was also calculated for studies where data reporting permitted this. Standard error (SE) was calculated and used to formulate 95% confidence intervals (CI).

Sensitivity and specificity of meta-analyses were performed using a random-effects model to calculate summary effect sizes with 95% confidence intervals. I^2^ was calculated to assess heterogeneity. For sensitivity meta-analysis, studies at high risk of bias were excluded. As specificity could only be calculated in five studies, those at high risk of bias were not excluded from specificity of meta-analysis. For studies where both sensitivity and specificity could be calculated, the relationship between these values was assessed using Pearson’s correlation coefficient. Visual assessment of publication bias was performed using a funnel plot.

## Results

**Figure 1.**
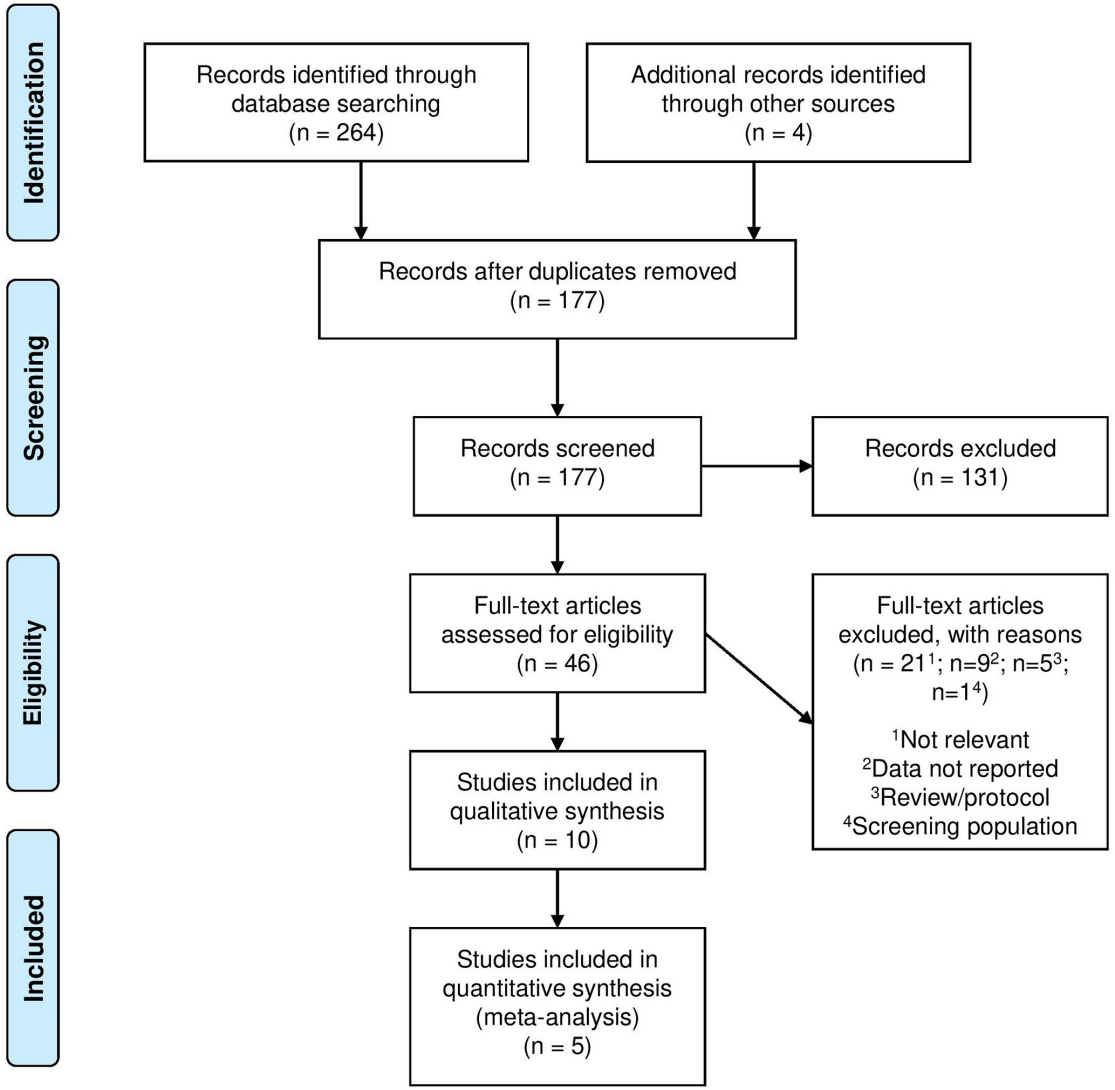
PRISMA flow diagram.

Initial database search revealed 173 items excluding duplicates. Four further items were identified through other sources (grey literature). Screening of title and abstract led to 46 items that were identified for full-text review. Thirty-six did not meet inclusion criteria as either the population was not representative, necessary data were not reported, radiographs were used for screening, or items were reviews or protocols. Ten studies were included for review.^19–28^ See Figure 1 for the PRISMA inclusion flowchart.

Included articles were published between 2006 and 2018, so are likely to represent contemporary practice and technology. All studies were performed in the UK or Northern Europe, so populations are likely to be comparable. Four studies were performed prospectively and six retrospectively. Four studies reported pathological diagnosis while six relied on radiological diagnosis. Diagnostic accuracy of chest radiographs stated aim of the study in only one case. All studies were set either in primary-care practices or acute general hospitals with data collected from referrals from primary care. Range of mean ages for each study was 53 to 71, with median 66 and interquartile range (IQR) 8. Full study details can be found in Table 1.

**Table 1.** Study details

Year	Authors	Journal	Setting	Country	Study type	Population	Mean age	Male (%)	N	CXRs (n)	Diagnosis	Sensitivity (95% CI)	Specificity	Risk of bias^*a*^
2006	Bjerager et al.	The British Journal of General Practice	Primary care	Denmark	Retrospective	Patients diagnosed with lung cancer in primary care	66	64	84	58	NR	79.31% (66.65% to 88.83%)	U	Concerns
2006	Stapley et al.	The British Journal of General Practice	Primary care	England	Retrospective	Patients diagnosed with lung cancer in primary care	71	69	247	164	Pathological	76.83% (69.61% to 83.05%)	U	Concerns
2006	Speets et al.	The British Journal of General Practice	Primary care	Netherlands	Prospective	Patients referred for CXR from primary care	57	53	870	142	NR	100% (71.51% to 100.00%)	U	High
2012	Harris et al.	Lung Cancer	Acute general hospital	England	Retrospective	Patients referred for CXR from primary care	NR	NR	798	797	NR	100.00% (98.31% to 100.00%)	U	High
2014	Ades et al.	International Journal of Epidemiology	Primary care	England	Retrospective	Patients identified from lung cancer registry with matched controls	NR	NR	1482	268	Radiological	91.02% (85.62% to 94.89%)	56.44% (46.20% to 66.28%)	Concerns
2014	Robinson et al.	European Respiratory Journal	Acute general hospital	England	Retrospective	Patients referred for TWR from primary care	NR	NR	143	143	NR	72.5% (56.11% to 85.40%)	60.19% (50.08% to 69.71%)	High
2015	Neal et al.	Primary Health Care Research & Development	Primary care	Wales	Retrospective	Patients diagnosed with lung cancer	69	NR	118	79	Pathological	70.89% (59.58% to 80.57%)	U	High
2017	Neal et al.	British Journal of Cancer	Primary care	Wales and England	Prospective	Patients referred for CXR from primary care	68	51	255	250	Pathological	75% (19.41% to 99.37%)	99.19% (97.09% to 99.90%)	Low
2017	Kedgley et al.	Lung Cancer	Thoracic medicine clinic in acute hospital	England	Retrospective	Patients referred for TWR from primary care	64	NR	56	38	NR	76.92% (46.19% to 94.96%)	40.00% (21.13% to 61.33%)	High
2018	Woznitza et al.	Clinical Radiology	Acute general hospital	England	Prospective	Patients referred for CXR from primary care	53	NR	1687	105	NR	80.00% (44.39% to 97.48%)	82.11% (72.90% to 89.22%)	Concerns

CXR, chest radiograph; NR, not reported; U, unable to calculate.

a Overall risk, synthesised using QUADAS-2 tool.

**Figure 2.**
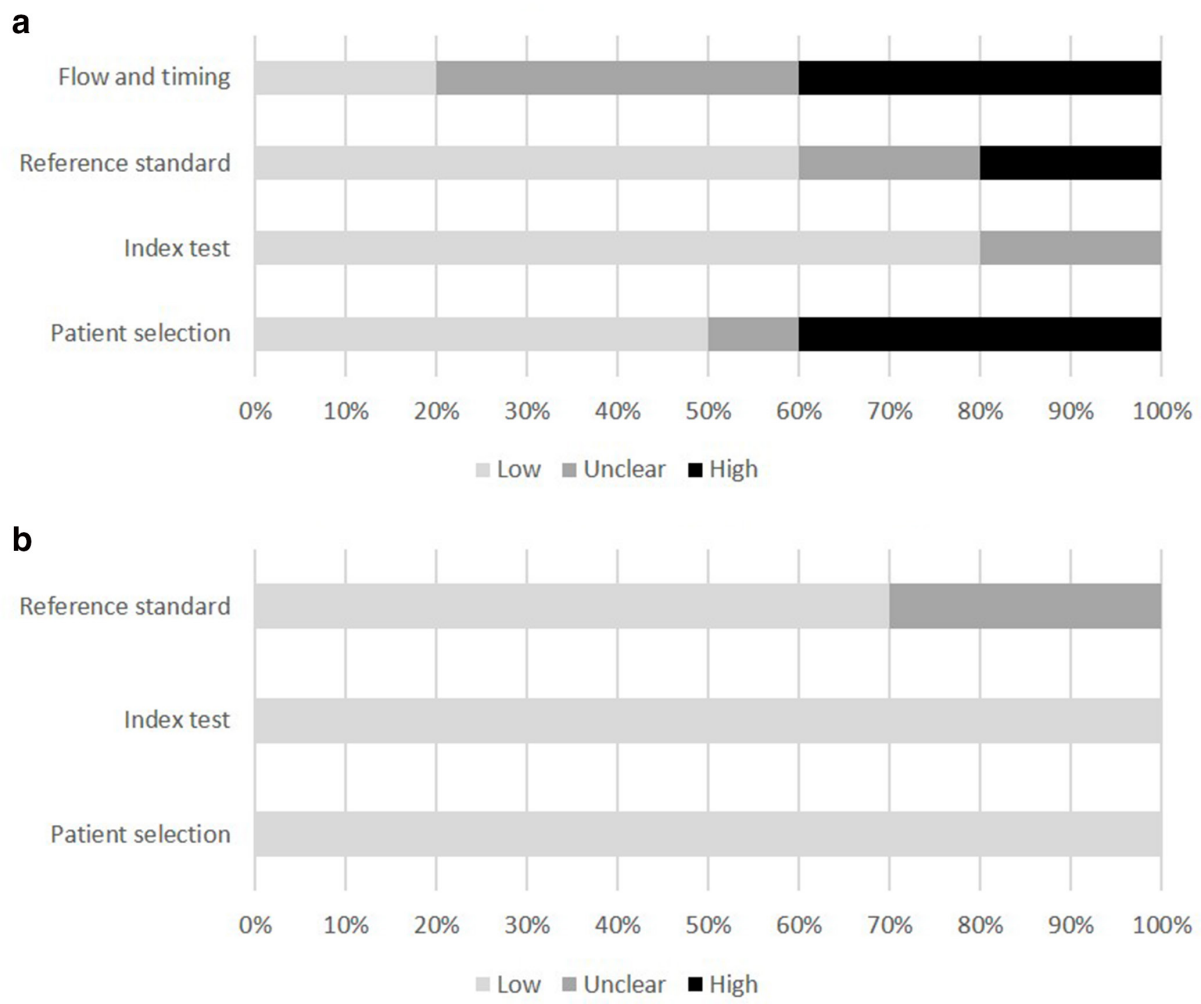
(a) Proportion of studies with low, unclear, or high risk of bias (b) Proportion of studies with low, unclear, or high concerns regarding applicability.

Five studies were judged to be at overall high risk of bias using theQUADAS-2 tool. Four studies had some concerns about bias, and one study was judged low risk of bias. Figure 2 demonstrates the proportions of studies and their risk of bias in each of the domains assessed by QUADAS-2. See Supplementary Material 1 for risk of bias assessment for each domain in each study.

**Figure 3.**
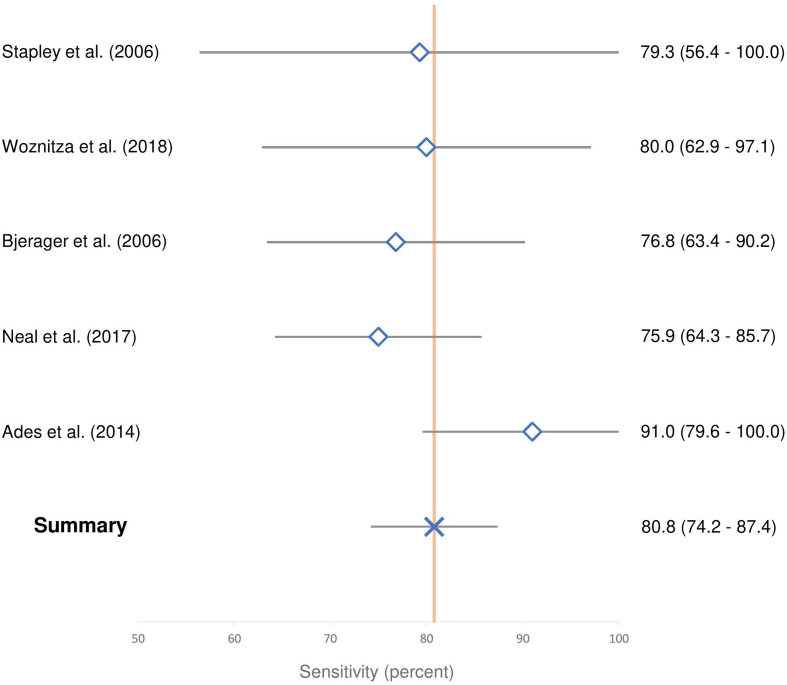
Forest plot demonstrating study and summary sensitivity, with 95% confidence intervals.

**Figure 4.**
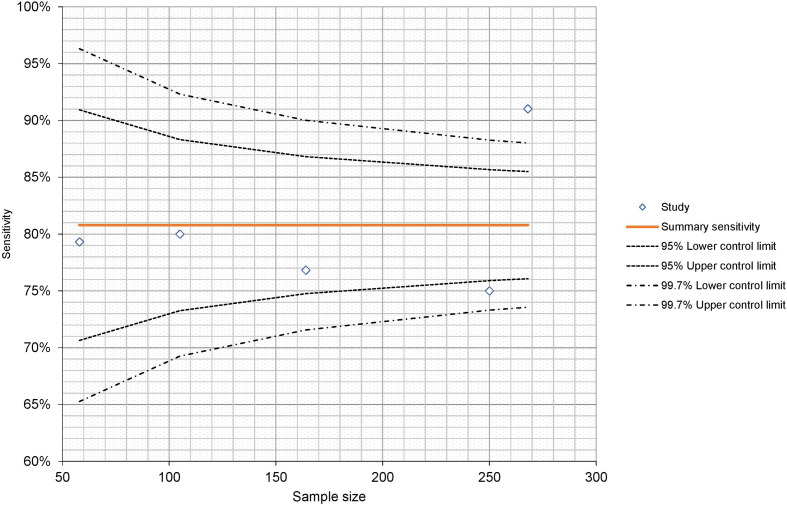
Funnel plot demonstrating study sample size against calculated sensitivity.

The five studies that were not at high risk of bias were assessed for conceptual homogeneity and, on being judged sufficiently homogeneous, were included for sensitivity meta-analysis.^19,20,23,26,28^ Summary sensitivity was 81% (SE 3.4%, CI 74 to 87%). I^2^ was zero, indicating no observed heterogeneity and appropriate selection of articles for meta-analysis.^29^ These data are displayed graphically as a Forest plot in Figure 3. Visual assessment of risk of publication bias performed using funnel plot (Figure 4) suggested a low risk of publication bias.

Specificity could be calculated in five studies, three of which were not at high risk of bias. Summary specificity was 68% (SE 9.9%, CI 49 to 87%). I^2^ was 6.7, indicating some heterogeneity.

Sensitivity and specificity could both be calculated in five studies. Pearson’s correlation coefficient between these values was −0.21.

## Discussion

### Summary

This systematic review and meta-analysis suggest the chest radiograph, performed in primary care for symptomatic individuals, has a sensitivity of 81% (CI 74–87%) and specificity of 68% (CI 49–87%). Evidence for sensitivity is strong due to selection of studies that were not at high risk of bias, low heterogeneity between studies, and low risk of publication bias. Evidence for specificity is weaker due to heterogeneous study design and variance between reported outcomes. There is evidence for a threshold effect between studies, as a negative correlation exists between sensitivity and specificity (*r* = −0.21), indicating that a higher sensitivity is associated with reduced specificity and vice versa, possibly due to differences in patient selection.

This information is valuable to primary-care clinicians investigating patients with symptoms suspicious for lung malignancy. Chest radiograph remains the first-line investigation in this scenario, and we have demonstrated a false-negative rate of just under 20%. This suggests nearly one-fifth of patients with lung malignancy may receive false reassurance following a negative chest radiograph. As such, if a high clinical suspicion for lung malignancy is held, follow-up, referral to secondary care, or further imaging should be considered.

This information will also be valuable to policymakers, as primary-care physicians often lack direct access to further imaging modalities, such as computed tomography. Existing literature suggests that as cross-sectional imaging has higher sensitivity than plain film radiography, its use in primary care for high-risk patients can reduce time to diagnosis of lung malignancy.^30^ However, these benefits must be weighed with the increased risks of radiation exposure and cost of investigation. Further research must be conducted to establish the performance of cross-sectional imaging in a symptomatic primary-care population, and randomised prospective methodology would provide high-quality evidence to inform these decisions.

### Strengths and limitations

This study employed a comprehensive search strategy and all records identified by the initial search were screened by title and abstract to minimise the likelihood of inappropriate exclusion. Appropriate application of inclusion and exclusion criteria led to a final selection of studies that was targeted towards and representative of a primary-care setting. Articles were all published within the last 15 years, suggesting they will be representative of contemporary technology and practice. Furthermore, a subselection of included studies was of sufficient quality and homogeneity to perform meta-analysis.

There were, however, limitations to this study. Although the search strategy was comprehensive, there remains a risk of incomplete record retrieval and reporting bias. Five of the studies were judged at high risk of bias, using the QUADAS-2 tool, and of the studies incorporated into meta-analysis, only one was at low risk of bias, the rest having some concerns. Furthermore, there was heterogeneity in study design, which limits comparability between studies, beyond those used for meta-analysis. Furthermore, as interpretation of chest radiographs is dependent on technical features such as the quality of the image, as well as reporter expertise, there is further heterogeneity introduced as these variables could not be standardised between studies.

Meta-analyses risk overstating summary statistics due to risk of publication bias where smaller studies with lower effect sizes are more likely to be published than smaller studies with greater effect sizes. Visual assessment of risk of publication bias using funnel plot suggests there is low risk of publication bias, as the plot is overall symmetrical and there does not appear to be a lack of smaller studies reporting lower sensitivities. As sensitivity of chest radiograph was not the primary outcome measure in any of the studies included for meta-analysis, there is a lower likelihood that publication bias has led to overstatement of summary sensitivity.

Similarly, it is important to note that none of the studies conformed to usual standards of investigations for diagnostic accuracy. The QUADAS-2 tool demonstrated that in several studies risk of bias was introduced due to an inadequate interval between the chest radiograph and the detection of malignancy, as well as attrition of participants. This highlights a need for prospective studies performed in a primary-care setting with unselected, representative populations and appropriate follow-up periods to provide an accurate confirmation of the sensitivity of chest radiograph to detect lung malignancy.

### Comparison with literature

Our findings are consistent with a previous systematic review, which has suggested an estimate of 77 to 80% for the sensitivity of chest radiograph to detect lung malignancy in symptomatic patients.^5^ This review did not focus on primary care, and only two studies were performed in a primary-care setting. These studies were also included in our review and analysis.^19,20^ As this article reproduces these findings, it adds strength to this estimate, and we agree with Bradley and colleagues (2019) that further work is required to investigate which patients can be reassured by a negative chest radiograph and which require further follow-up or cross-sectional imaging. One possibility is the utilisation of risk algorithms, as suggested by Kedgley and colleagues (2017);^27^ however, further work is required to identify the populations and scenarios in which they can be safely applied. Any strategies used to attenuate the relatively poor performance of this investigation for such a significant pathology must weigh the risks of false reassurance with the potential financial and physical costs of over-investigation, and prospective randomised trials with long-term follow-up may help identify where the balance of risk lies.

## Conclusions

The sensitivity of chest radiograph to detect lung malignancy in symptomatic patients presenting to primary care is 81%, with specificity 68%. This implies a high risk of false-negative, with potential for catastrophic consequences. Primary-care physicians should not be reassured by a negative chest radiograph if they have a high clinical suspicion for malignancy and should consider cross-sectional imaging or secondary-care referral. Policymakers should incorporate this information into national strategy, and consider widening access to cross-sectional imaging modalities for primary-care physicians. This must be weighed up with potential cost and the risks of increased radiation and contrast exposure. Future work should focus on large, well-conducted and well-reported, prospective studies designed to compare the performance of chest radiograph with cross-sectional imaging in symptomatic primary-care populations, distinguishing patients who can be reassured with a negative radiograph from those that should be investigated further, for example, with risk stratification tools.
